# Deregulated c-Myc requires a functional HSF1 for experimental and human hepatocarcinogenesis

**DOI:** 10.18632/oncotarget.21469

**Published:** 2017-10-03

**Authors:** Antonio Cigliano, Maria G. Pilo, Lei Li, Gavinella Latte, Marta Szydlowska, Maria M. Simile, Panagiotis Paliogiannis, Li Che, Giovanni M. Pes, Giuseppe Palmieri, Maria C. Sini, Antonio Cossu, Alberto Porcu, Gianpaolo Vidili, Maria A. Seddaiu, Rosa M. Pascale, Silvia Ribback, Frank Dombrowski, Xin Chen, Diego F. Calvisi

**Affiliations:** ^1^ Institut für Pathologie, Universitätsmedizin Greifswald, Greifswald, Germany; ^2^ Department of Clinical and Experimental Medicine, University of Sassari, Sassari, Italy; ^3^ School of Pharmacy, Tongji Medical College, Huazhong University of Science and Technology, Wuhan, Hubei, China; ^4^ Department of Bioengineering and Therapeutic Sciences and Liver Center, University of California, San Francisco, CA, USA; ^5^ Institute of Biomolecular Chemistry, National Research Council, Sassari, Italy; ^6^ Unit of Pathology, Azienda Ospedaliero Universitaria Sassari, Sassari, Italy

**Keywords:** hepatocellular carcinoma, liver cancer, HSF1, c-Myc, signalling pathways

## Abstract

Deregulated activity of the c-Myc protooncogene is a frequent molecular event underlying mouse and human hepatocarcinogenesis. Nonetheless, the mechanisms sustaining c-Myc oncogenic activity in liver cancer remain scarcely delineated. Recently, we showed that the mammalian target of rapamycin complex 1 (mTORC1) cascade is induced and necessary for c-Myc dependent liver tumor development and progression. Since the heat shock factor 1 (HSF1) transcription factor is a major positive regulator of mTORC1 in the cell, we investigated the functional interaction between HSF1 and c-Myc using *in vitro* and *in vivo* approaches. We found that ablation of *HSF1* restrains the growth of c-Myc-derived mouse hepatocellular carcinoma (HCC) cell lines, where it induces downregulation of c-Myc levels. Conversely, silencing of *c-Myc* gene in human and mouse HCC cells led to downregulation of HSF1 expression. Most importantly, overexpression of a dominant negative form of HSF1 (HSF1dn) in the mouse liver via hydrodynamic gene delivery resulted in the complete inhibition of mouse hepatocarcinogenesis driven by overexpression of c-Myc. Altogether, the present results indicate that a functional HSF1 is necessary for c-Myc-driven hepatocarcinogenesis. Consequently, targeting HSF1 might represent a novel and effective therapeutic strategy for the treatment of HCC subsets with activated c-Myc signaling.

## INTRODUCTION

Liver cancer is the sixth most common tumor and the second most frequent cause of cancer-related mortality worldwide [1, 2]. Hepatocellular carcinoma (HCC) is the predominant liver cancer subtype, accounting for ∼90% of all cases [1, 2]. Unlike many other tumor entities, HCC incidence will rise considerably in the coming years and has already doubled in the last two decades [1, 2]. Mortality will also increase in parallel, partly due to the lack of effective therapies against this deadly neoplasm [1, 2]. Indeed, partial liver resection and liver transplantation are effective treatments against HCC, but they can be applied only in the early phases of the disease [3]. Furthermore, the multi-kinase inhibitor sorafenib, the only FDA-approved targeted therapy for advanced HCC patients, confers a survival benefit of only 2-3 months on average [3]. As a consequence, new therapeutic strategies should be developed in order to significantly improve the prognosis of HCC patients. For this purpose, the identification of new molecular targets is imperative. The ideal target should be selected based on the fact that its modulation would affect only the survival of cancer cells, while sparing the normal ones. In this regard, one option would be to target cancer driver genes, which actively sustain carcinogenesis and whose inhibition triggers tumor restraint. However, many cancer driver genes are not easily druggable. A feasible alternative would be to target key downstream effectors of driver genes, whose repression would be equally deleterious for tumor cells.

Among the putative candidates for targeted therapies in HCC is the c-Myc protooncogene, which is frequently over-expressed and/or amplified in HCC and whose levels directly correlate with the poor outcome of HCC patients [4-8]. c-Myc is a multifunctional, nuclear phosphoprotein that belongs to the basic helix loop helix leucine zipper family [4-5]. Previously, it has been demonstrated that c-Myc contributes to malignant transformation and tumor progression by regulating the expression of a large number of genes involved in cell growth, differentiation, mitochondrial biogenesis, metabolism, carcinogenesis, and stem cell self-renewal [4-5]. In mouse models of liver cancer, it has been shown that aberrant overexpression of c-Myc is sufficient to induce HCC development [8-9], while c-Myc downregulation rapidly triggers tumor dormancy and regression [8]. In addition, transcriptomic analysis of a human HCC cohort identified c-Myc as a driver gene for malignant conversion of liver dysplastic nodules into early HCC [10]. Furthermore, a classifier constructed from c-Myc target genes was successful in robustly discriminating early HCC from high-grade and low-grade dysplastic nodules [10].

Despite this body of evidence, the molecular mechanisms whose inactivation impairs c-Myc driven hepatocarcinogenesis remain poorly delineated. Also, *c-Myc* is not an easy druggable gene, as it does not possess targetable domains [11]. In a previous study, we showed that c-Myc requires an intact mammalian target of rapamycin (mTOR) pathway in order to exert its oncogenic potential in the liver [12]. mTOR is an evolutionary conserved pathway composed of two distinct complexes, referred to as complex 1 and complex 2 (mTORC1 and mTORC2) [12, 13]. Once activated, mTOR regulates multiple functions of the cell, including proliferation, metabolism, and survival [13, 14]. Recently, we and others have found that the heat shock factor 1 (HSF1) is necessary for sustaining the activity of the mTOR pathway or *vice versa* in mouse and human hepatocarcinogenesis as well as in many other tumor types [15-21]. In addition, it has been shown that HSF1 depletion strongly reinforces apoptosis in mouse embryonic fibroblasts overexpressing c-Myc [16]. HSF1 is a multifaceted transcription factor regulating the cell response to stressors as well as many cellular processes, including proliferation, apoptosis, and metabolism [21-27].

In the present study, we investigated the functional interplay between HSF1 and c-Myc. In particular, we addressed the role of HSF1 on c-Myc-induced hepatocarcinogenesis via *in vitro* and *in vivo* approaches. We found that HSF1 is strongly upregulated in c-Myc mouse lesions and cell lines, where its inactivation completely suppresses liver tumor development and robustly restrains cell growth. Based on the notion that potent HSF1 inhibitors have been discovered and developed, the present data suggest that HSF1 might represent a valuable, druggable target in human HCC subsets characterized by the activation of the c-Myc protooncogene.

## RESULTS

### HSF1 pathway is activated and required for c-Myc driven hepatocarcinogenesis

**Figure 1:**
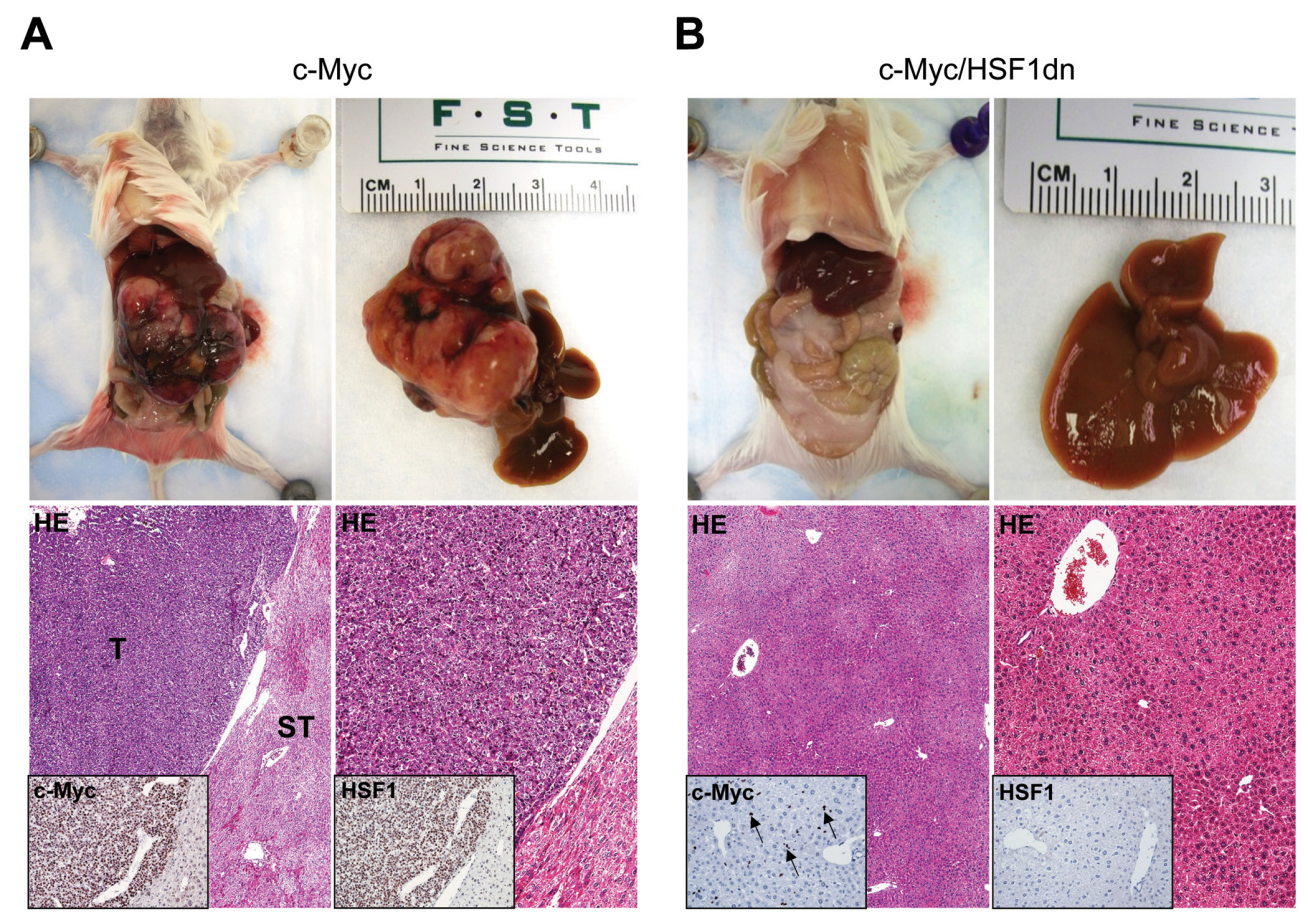
Inactivation of HSF1 abolishes c-Myc dependent hepatocarcinogenesis in mice (**A.** *upper panels*) Overexpression of *c-Myc* promotes the development of multiple liver tumors by 6 weeks post hydrodynamic injection in mice (indicated as c-Myc). Macroscopically, livers of c-Myc mice appeared enlarged, and characterized by the presence of numerous nodules occupying most of its surface. Areas of hemorrhages were also present. (*A; lower left panel*) Microscopically, the liver parenchyma of c-Myc mice was occupied by large hepatocellular tumors (T) compressing the surrounding non-neoplastic liver tissues (ST). (*A; lower right panel*) Hepatocellular tumors were mainly composed of small, malignant cells with a basophilic cytoplasm, as evidenced at higher magnification. Importantly, tumors displayed a homogeneous nuclear immunoreactivity for c-Myc and HSF1 (*insets*). (**B.** *upper panels*) In striking contrast, overexpression of a dominant negative form of HSF1 (*HSF1dn*) together with *c-Myc* (indicated as c-Myc/HSF1dn) completely inhibits hepatocarcinogenesis at the same time point in mice. Livers of c-Myc/HSF1dn mice appeared normal macroscopically, and showed the absence of preneoplastic and neoplastic lesions microscopically (*lower left panel*). No altered hepatocytes were detected at higher magnification (*lower right panel*). Scattered hepatocytes exhibited nuclear immunolabeling for c-Myc (some are indicated by arrows), whereas HSF1 staining was very faint or completely absent (*insets*). Original magnifications: 40X and 100x. Abbreviation: HE, hematoxylin and eosin.

**Figure 2:**
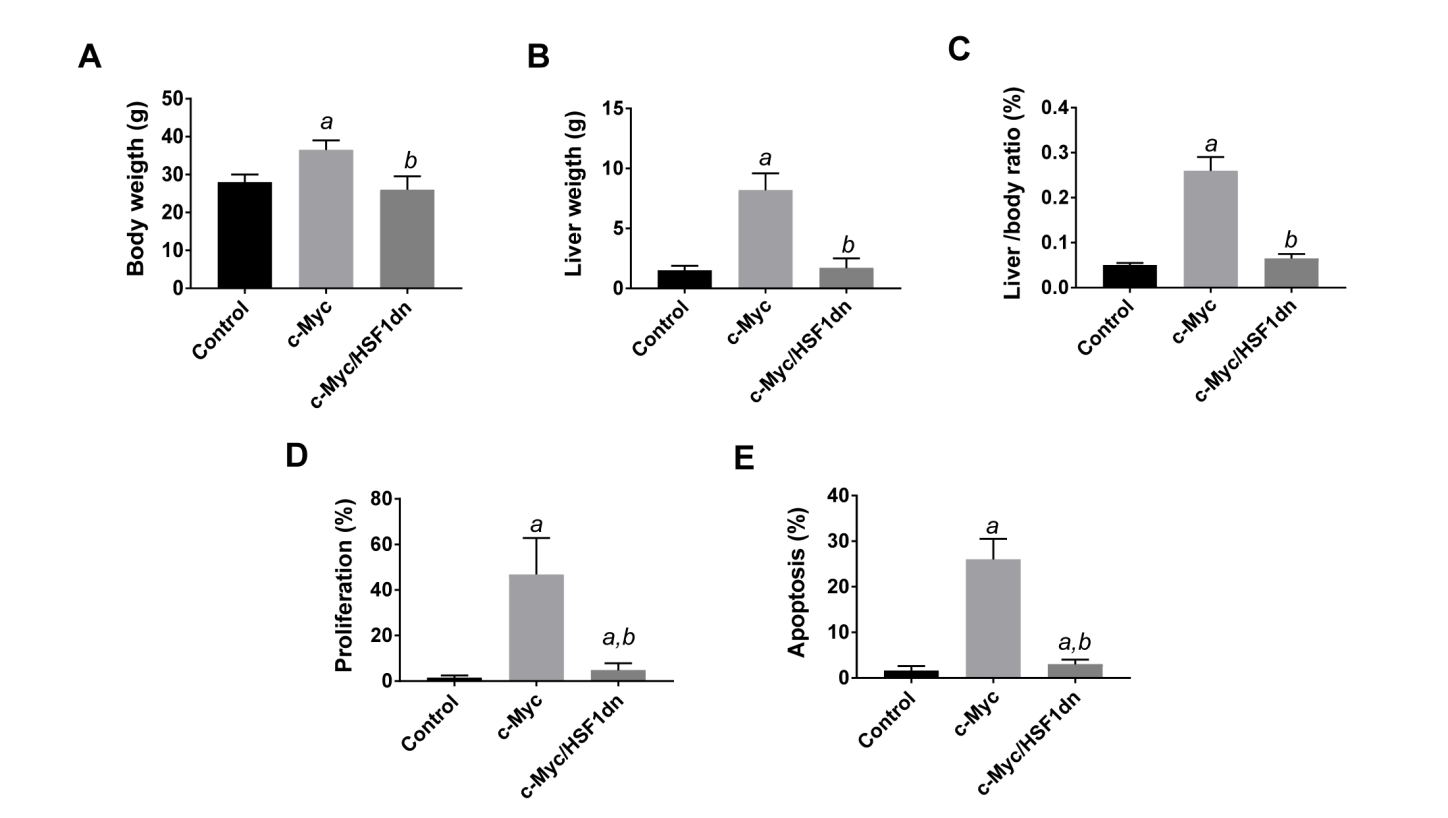
Suppression of hepatocarcinogenesis following HSF1 inactivation is accompanied by normalization of body, liver, and cellular parameters in mice Tukey–Kramer test: *P* < 0.0001 *a*, vs control (mice injected with empty vector); *b*, vs c-Myc mice. Ten mice per each group were analyzed.

To investigate whether HSF1 is required for c-Myc driven hepatocarcinogenesis, we overexpressed c-Myc while simultaneously inactivating HSF1 in the hepatocytes. To achieve this goal, we hydrodynamically transfected FVB/N mice with c-Myc and a dominant negative form of HSF1 [28] (HSF1dn; these mice will be referred to as c-Myc/HSF1dn mice; *n* = 20). As a control, we hydrodynamically transfected FVB/N mice with either empty vector (referred to as control mice; *n* = 10) or c-Myc (referred to as c-Myc mice; *n* = 10). All c-Myc mice showed an enlarged liver with a consequent increase in body and liver weight as well as in liver to body weight ratio when compared to control mice, and required to be euthanized within 6 weeks post hydrodynamic injection due to high tumor burden (Figure 1A, upper panels; Figure 2A-2C). In striking contrast, none of the c-Myc/HSF1dn mice (*n* = 5) exhibited abdomen enlargement (Figure 1B, upper panels) or increased body and liver weight when compared with control mice and c-Myc mice at the same time point (Figure 2A-2C). At the cellular level, augmented liver weight was paralleled by increased proliferation and apoptosis in c-Myc mice when compared with control and c-Myc/HSF1dn mice (Figure 2D, 2E). Histologically, most of the liver parenchyma of c-Myc mice was occupied by multiple, colliding hepatocellular tumors (Figure 1A, lower panels), in accordance with previous reports [8, 12]. In striking contrast, none of the c-Myc/HSF1dn mice showed the presence of preneoplastic and neoplastic lesions at 6 weeks post-injection (Figure 1B, lower panels). Indeed, livers from c-Myc/HSF1dn mice appeared completely normal at the histological level (Figure 1B, lower panels). Subsequently, additional groups of c-Myc/HSF1dn mice (*n* = 5 for each time point) were harvested 18 and 36 weeks post hydrodynamic injection. Once again, no abdomen enlargement, increased in body and liver weight, or liver histological alterations were detected in c-Myc/HSF1dn mice at the remaining time points examined (data not shown).

**Figure 3:**
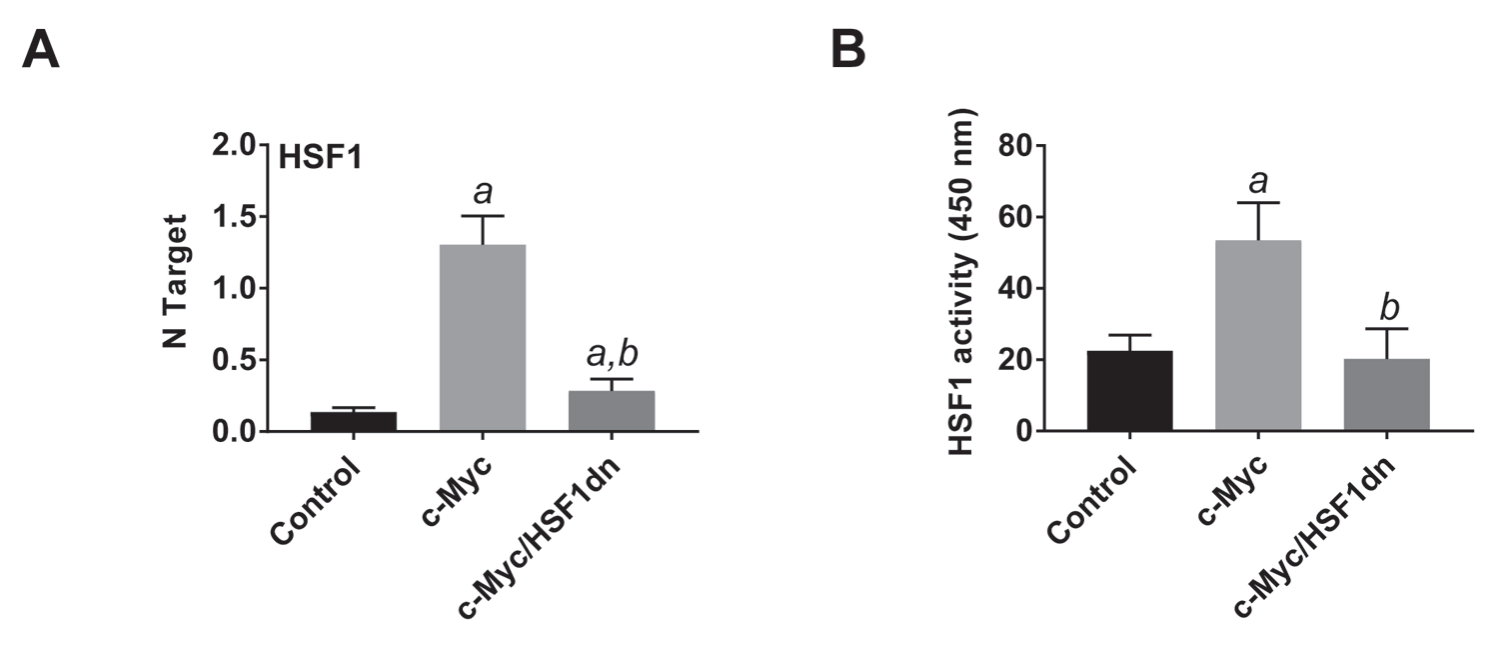
HSF1 is overexpressed and strongly activated in c-Myc mice **A.** HSF1 mRNA levels as assessed by reverse-transcription real-time PCR. Number target (NT) = 2^-ΔCt^, wherein ΔCt value of each sample was calculated by subtracting the average Ct value of the *HSF1* gene from the average Ct value of the *β-actin* gene. **B.** HSF1 activity, as determined using the HSF1 ELISA kit (Enzo Life Sciences), following the manufacturers’ instructions. Tukey–Kramer test: *P* < 0.0001 *a*, vs control (mice injected with empty vector); *b*, vs c-Myc mice. Ten mice per each group were analyzed.

**Figure 4:**
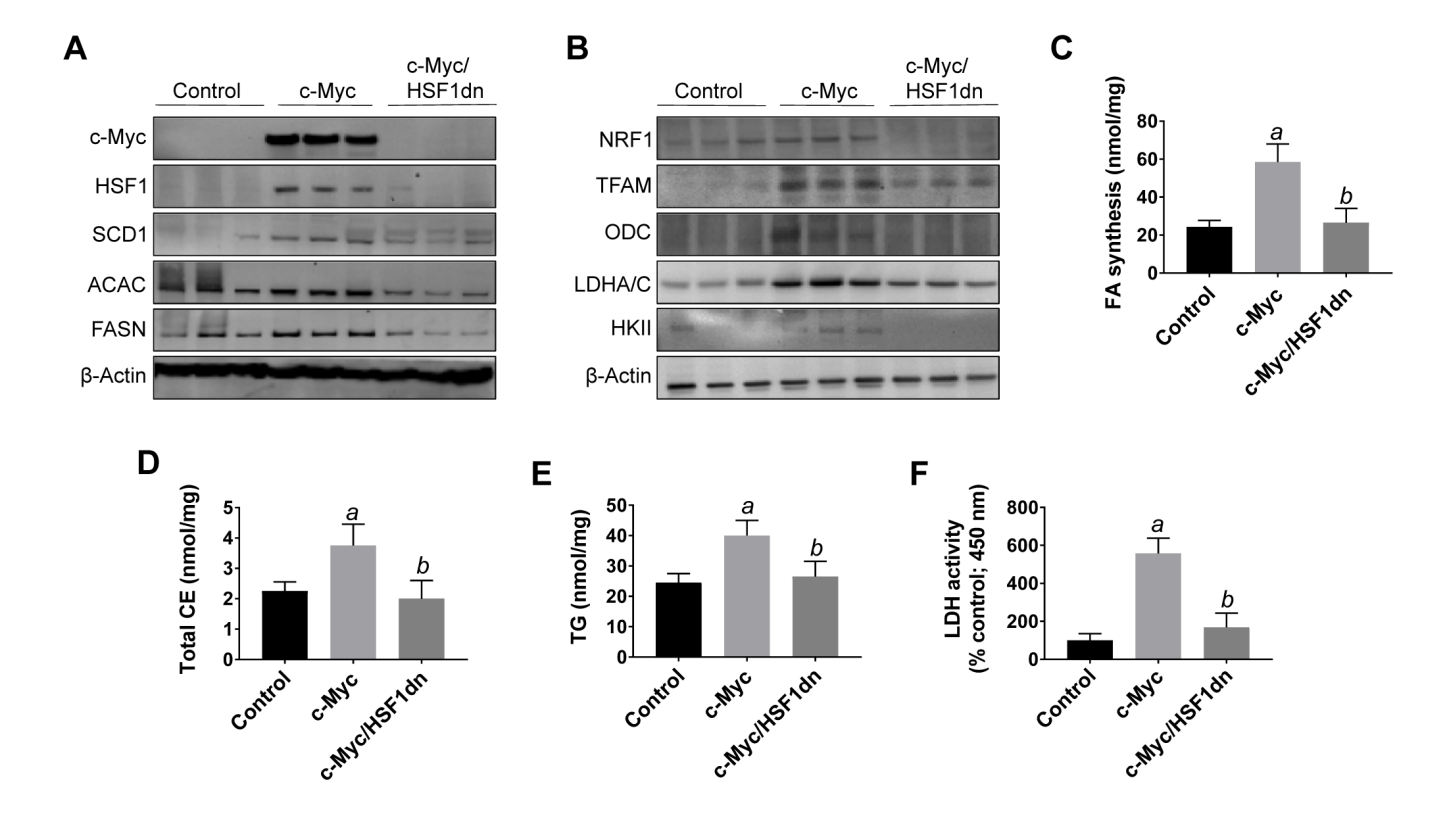
Suppression of hepatocarcinogenesis following HSF1 inactivation is accompanied by downregulation of c-Myc and its downstream effectors in the mouse liver **A.**, **B.** Representative Western blot analysis of livers from control mice (injected with empty vector), c-Myc mice (injected with *c-Myc*), and c-Myc/HSF1dn mice (co-injected with *c-Myc* and *HSF1dn*). Inactivation of HSF1 in c-Myc/HSF1dn mice resulted in downregulation of c-Myc. Levels of c-Myc downstream effectors (FASN, ACAC, SCD1, ODC, TFAM, NRF1, HKII, LDHA/C) were downregulated in c-Myc/HSF1dn mice when compared with c-Myc mice. At least five livers from each group of mice were used for the analysis, and representative images are shown. β-Actin was used as a loading control. **C.**-**F.** Fatty acid biosynthesis, total cholesterol and triglyceride content, and lactate dehydrogenase activity were strongly induced in the livers of c-Myc mice, and equally lower in control and c-Myc/HSF1dn livers. Tukey–Kramer test: *P* < 0.0001 *a*, vs control (mice injected with empty vector); *b*, vs c-Myc mice. Abbreviations: FA, fatty acid; CE, cholesterol; TG, triglycerides; LDH, lactate dehydrogenase.

To investigate the possible crosstalk between c-Myc and HSF1 pathways in hepatocarcinogenesis, we evaluated the status of HSF1 in c-Myc mouse tumor tissues. Of note, we found that HSF1 was strongly upregulated in c-Myc tumor samples (Figures 1 and 3). Specifically, a strong and diffuse nuclear immunoreactivity for c-Myc and HSF1 characterized the lesions of c-Myc mice (Figure 1A, lower panel insets). In addition, high HSF1 mRNA and protein levels as well as elevated HSF1 activity were detected in c-Myc lesions (Figures 3, 4). In striking contrast, absent or very faint immunolabeling for c-Myc and HSF1 was observed in the livers from c-Myc/HSF1dn mice (Figure 1B, lower panel insets). Consistently, c-Myc/HSF1dn livers displayed low levels of HSF1 mRNA, protein, and activity that were equivalent to those detected in control livers (Figures 3, 4). At the molecular level, c-Myc/HSF1dn mice showed downregulation of HSF1 and c-Myc as well as c-Myc targets involved in *de novo* lipogenesis (fatty acid synthase or FASN; acetyl-CoA carboxylase or ACAC; and stearoyl-CoA desaturase 1 or SCD1), mitochondrial biogenesis (nuclear respiratory factor 1 or NRF1; and transcription factor A or TFAM), polyamine metabolism (ornithine decarboxylase or ODC), and glycolysis (lactate dehydrogenease A/C or LDHA/C; and hexokinase II or HKII) (Figure 4A, 4B). As expected, the same c-Myc targets were strongly induced in c-Myc liver lesions (Figure 4A, 4B).

To further confirm the effects of HSF1 silencing on c-Myc-overexpressing livers, we assayed the levels of fatty acid biosynthesis, total cholesterol and triglycerides levels in livers from control, c-Myc, and c-Myc/HSF1dn livers at 6 weeks post-hydrodynamic gene delivery (Figure 4). As expected, c-Myc liver specimens showed the highest levels of fatty acid biosynthesis, total cholesterol and triglycerides, whereas similar levels were detected in control and c-Myc/HSF1dn mice (Figure 4C-4E). In addition, levels of glycolysis, as assessed by LDH activity were highest in c-Myc mice and equivalent in control and c-Myc/HSF1dn mice (Figure 4F).

Altogether, the present results indicate that HSF1 is activated and required for c-Myc induced liver tumor formation in mice.

### Suppression of HSF1 reduces the levels of c-Myc and the *in vitro* growth of c-Myc mouse cell lines

**Figure 5:**
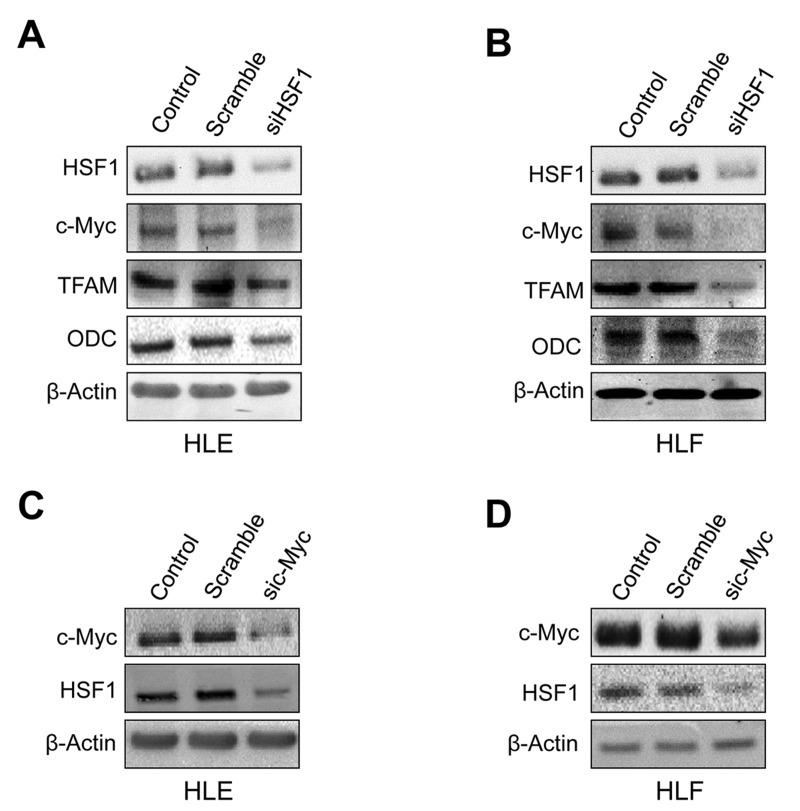
Mutual regulation of HSF1 and c-Myc in human HLE and HLF HCC cell lines **A.** Suppression of *HSF1* expression by specific siRNA for 48h induces downregulation of c-Myc and TFAM and ODC targets in HLE cells, as assessed by Western blot analysis. **B.** Equivalent results were obtained in HLF cells. **C.** Knockdown of c-Myc by siRNA for 48h triggers downregulation of HSF1 in HLE cells. **D.** Equivalent results were obtained in HLF cells. β-Actin was used as a loading control.

Next, we assessed the importance of HSF1 in regulating the levels of c-Myc in human HCC cell lines. For this purpose, the *HSF1* gene expression was knocked-down in HLE and HLF hepatoma cells with specific small interfering RNA (siRNA) (Figure 5, Supplementary Figure 1). Depletion of *HSF1* by siRNA resulted in strong reduction of proliferation and increase of apoptosis in the two cell lines (data not shown), in accordance with our previous report [15]. As hypothesized, we found that silencing of HSF1 reduced the protein levels of c-Myc and its targets TFAM and ODC in HLE and HLF cell lines (Figure 5A, 5B). Similarly, silencing of c-Myc reduced the levels of HSF1 in the same cells (Figure 5C, 5D). Equivalent results were obtained when assessing HSF1 and c-Myc mRNA levels (Supplementary Figure 1). Furthermore, treatment of the same cell lines with the HSF1 inhibitor KRIBB11 [29] resulted in decrease of c-Myc, TFAM, and ODC protein levels (Supplementary Figure 2). In addition, forced overexpression by transient transfection of HSF1 or c-Myc in the low-expressing MHCC97-L human HCC cell line led to the mutual upregulation of the other transcription factor (Supplementary Figure 3). Thus, this concordant body of data indicates a reciprocal, positive regulation by HSF1 and c-Myc in human liver cancer cells.

**Figure 6:**
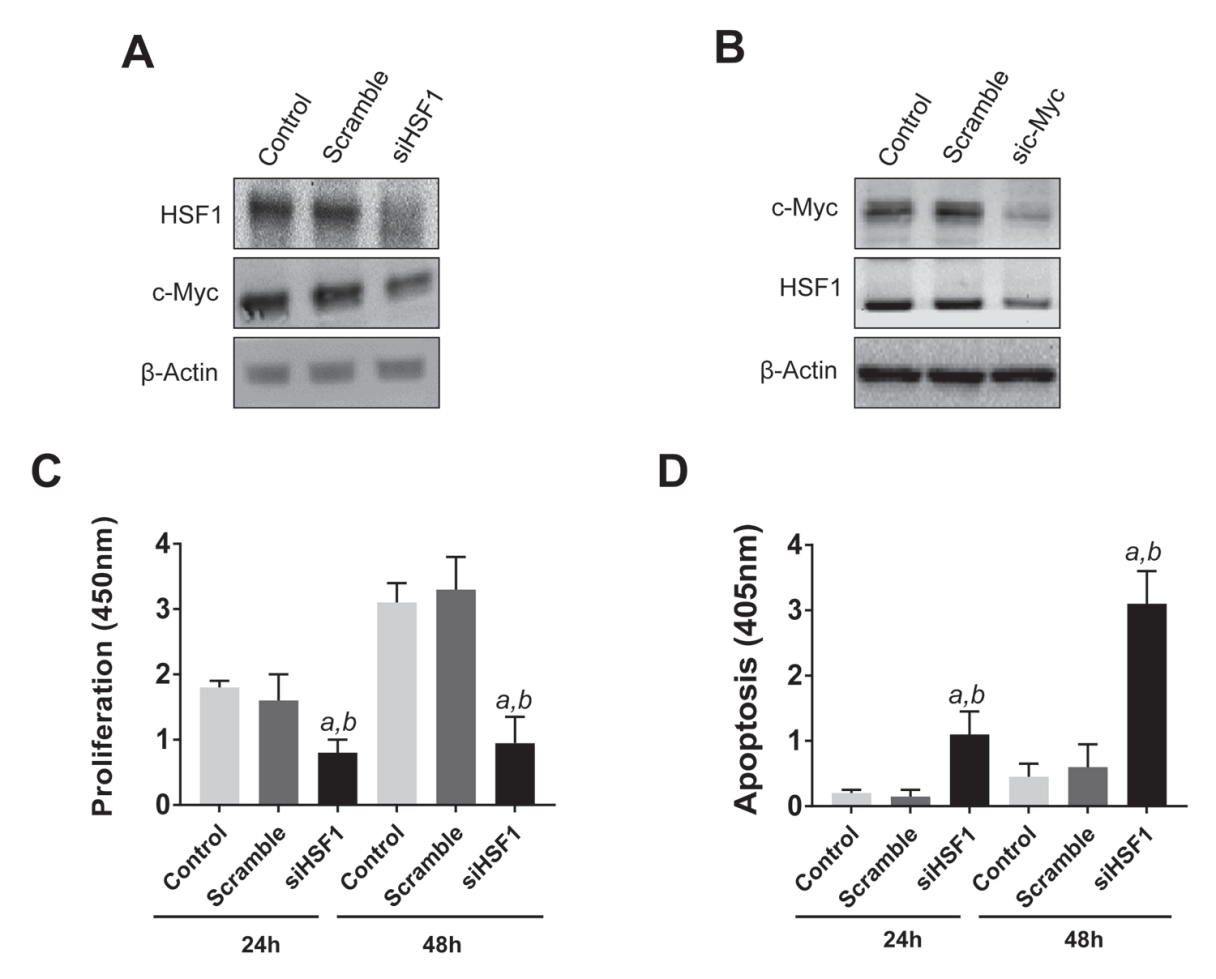
Reciprocal regulation of HSF1 and c-Myc and growth restraint following HSF1 silencing in the mouse HCC3-4 c-Myc cell line **A**. At the molecular level, HSF1 silencing leads to c-Myc downregulation, as assessed by Western blot analysis. **B**. Similarly, knockdown of c-Myc by siRNA triggers downregulation of HSF1. Equivalent results were obtained in HCC4-4 cells (Supplementary Figure 4). β-Actin was used as a loading control. **C**., **D**. Suppression of *HSF1* expression by specific siRNA for 24h and 48h results in decreased proliferation and augmented apoptosis. Tukey–Kramer test: *P* < 0.0001 a, vs control (untreated cells); b, vs scramble siRNA.

Subsequently, we evaluated the influence of HSF1 on the growth of c-Myc overexpressing cells. For this reason, the HCC3-4 and HCC4-4 HCC cell lines [29], isolated from c-Myc mouse liver tumors (overexpressing the human c-Myc gene), were subjected to HSF1 siRNA (Figure 6 and Supplementary Figures 4 and 5). Of note, a robust growth restraint followed HSF1 silencing in the HCC3-4 cell line, leading to reduced proliferation and increased apoptosis (Figure 6A, 6B). Once again, suppression of HSF1 expression led to significant downregulation of c-Myc and *vice versa* both at protein (Figure 6C, 6D) and mRNA level (Supplementary Figure 5). Equivalent results were obtained in the HCC4-4 cell line (Supplementary Figures 4 and 5). In accordance with these findings, treatment with the HSF1 inhibitor KRIBB11 triggered a pronounced, highly significant, decline in proliferation and increase of apoptotic cell death in HCC3-4 and HCC4-4 cells (Supplementary Figure 6).

Altogether, the present data indicate that inhibition of HSF1 is highly detrimental for the *in vitro* growth of c-Myc HCC cells, and HSF1 is a major regulator of c-Myc in human and murine HCC cell lines.

### HSF1 levels correlate with those of c-Myc in human HCC specimens

**Figure 7:**
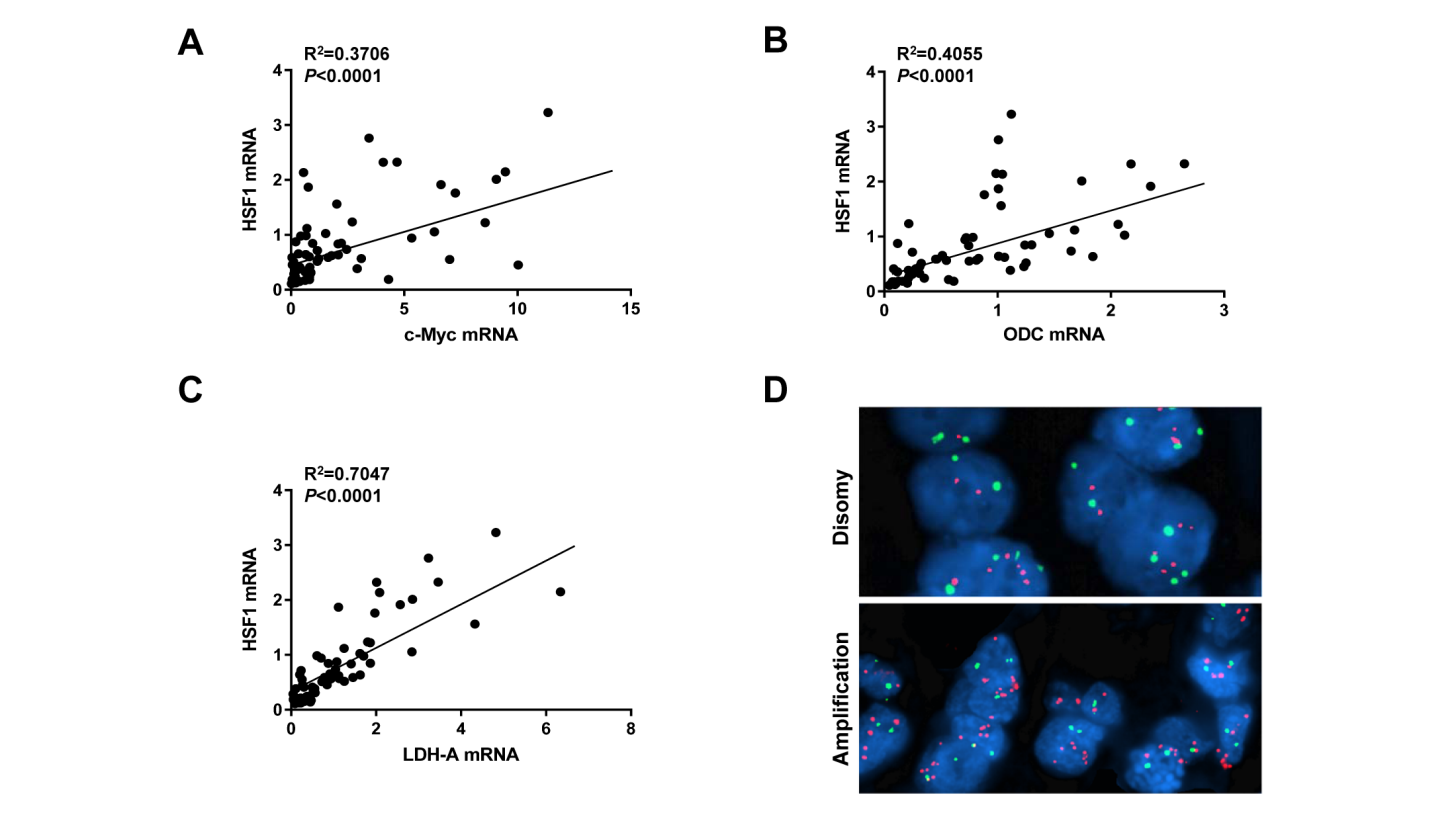
HSF1 and c-Myc mRNA levels positively correlate in human HCC specimens **A.** Relationship between *HSF1* and *c-Myc* expression levels; data were extracted from previous publications of our group and subjected to linear regression analysis [12, 15]. **B.**, **C.** A statistically significant, positive correlation was also found between mRNA levels of *HSF1* and those of two canonical c-Myc targets, namely *ODC* and *LDHA*. **D.** Example of *c-Myc* amplification patterns in HCC specimens as detected by fluorescence in situ hybridization (FISH). Upper panel: disomy (normal pattern); lower panel: *c-Myc* amplification.

Finally, we evaluated the relationship between HSF1 and c-Myc genes in human HCC. For this purpose, the *c-Myc* and *HSF1* mRNA levels data from previous publications from our group [12, 15] were related to each other. Of note, a significant, positive correlation between the mRNA levels of *HSF1* and those of *c-Myc* was revealed (Figure 7A). Similarly, mRNA levels of *HSF1* significantly and positively correlated with mRNA expression of two canonical c-Myc targets, namely *ODC* and *LDHA* in the same HCC samples (Figure 7B, 7C). Interestingly, when evaluating the amplification of *c-Myc* and *HSF1* genes in the same human HCC subset, we found that 8 of 64 (12.5%) and 24 of 64 (37.5%) HCC exhibited *c-Myc* and *HSF1* amplification, respectively (Figure 7). Importantly, only one HCC showed concomitant amplification of both *c-Myc* and *HSF1* genes, thus suggesting that these two genetic events are mutually exclusive along hepatocarcinogenesis. Furthermore, 22 of 32 (68.7%; *P* = 0.0023) HCC showing amplification of either *c-Myc* or *HSF1* belonged to the HCC subgroup with poorer prognosis, implying that these genetic alterations are associated with liver tumors with a biologically more aggressive phenotype.

Altogether, the present data indicate that HSF1 and c-Myc levels are directly correlated in human liver cancer.

## DISCUSSION

Aberrant activation of the c-Myc transcription factor due to overexpression, translocation, amplification, and/or protein stabilization has been detected in multiple tumor types, including HCC [4-8]. In the mouse liver, the oncogenic potential of c-Myc is underscored by its ability to induce tumor development when overexpressed and to abolish hepatocarcinogenesis when it is genetically inactivated [8, 9]. Consequently, c-Myc has been assumed to be a highly attractive target for the treatment of human HCC. In particular, it can be envisaged that c-Myc is an indispensable and non-redundant signaling hub for tumor initiation and maintenance, whose suppression might be difficult to be bypassed by HCC cells. Unfortunately, c-Myc itself is not an easily “druggable” protein, as it lacks enzymatic activity and does not possess any deep pocket, which could be eventually targeted by small molecule inhibitors [11]. For this reason, the identification of c-Myc co-factors and/or downstream effectors playing critical roles in tumor development or maintenance is of prime importance [11].

In the present study, we found that the HSF1 transcription factor is essential in supporting hepatocarcinogenesis driven by c-Myc. This assumption is based on the complete absence of preneoplastic and neoplastic liver lesions in c-Myc mice where HSF1 was inactivated as well as by the robust growth restraint induced in c-Myc mouse cell lines following HSF1 silencing. It is important to underline that equivalent results were obtained in human HCC cell lines and HCC samples, indicating that the functional interplay between c-Myc and HSF1 is conserved among species. As we recently showed that HSF1 depletion suppresses liver tumor development induced by the AKT protooncogene [15], the present evidence suggests that HSF1 might support liver malignant transformation and tumor progression driven by various oncogenes. In accordance with this hypothesis, it has been shown that genetic knockout of HSF1 impairs carcinogenesis in the mouse induced by mutant p53, oncogenic Ha-Ras, and platelet-derived growth factor B [16]. Nonetheless, a pilot study from our laboratory indicates that HSF1 depletion delays without suppressing hepatocarcinogenesis in AKT/Ras mouse preneoplastic and neoplastic lesions, which exhibit elevated HSF1 activity (Calvisi et al., unpublished observation). Even more intriguingly, we found that HSF1 inactivation does not affect Notch induced liver carcinogenesis in mice (Calvisi et al., unpublished observation). Although preliminary, these findings imply that some oncogenes are capable to drive the neoplastic process independent of HSF1. Additional studies are necessary to identify the oncogenes that are either affected or unaffected in their malignant potential by HSF1 modulation.

The molecular mechanism whereby HSF1 sustain c-Myc malignant properties remain to be better defined. Importantly, our data indicate that several c-Myc targets involved in *de novo* lipogenesis, glycolysis, polyamine metabolism, and mitochondrial biogenesis were concurrently downregulated in c-Myc mouse liver lesions following HSF1 inactivation. Further investigation is required to identify the pivotal players modulated by HSF1 in c-Myc dependent hepatocarcinogenesis. Nonetheless, we showed for the first time that HSF1 and c-Myc regulate each other’s levels, thus suggesting that HSF1 might suppress c-Myc dependent liver cancer development by directly acting on the *c-Myc* gene. On the other hand, numerous genes whose activity is -directly or indirectly- necessary for the survival of c-Myc overexpressing cells have been identified [11, 30-33]. Thus, we cannot exclude that HSF1 inactivation impairs c-Myc oncogenic potential by interfering with the activity of these genes. For instance, previous investigations have demonstrated that loss of *HMGCR* [29], *CDK9* [30], *ARK5* [31], or *CSNK1e* [32] gene is highly detrimental for the survival of cancer cells exhibiting c-Myc activation. However, preliminary data from our group show that suppression of HSF1 by siRNA or KRIBB11 has no effects on the levels of *CDK9*, *ARK5*, *HMGCR*, or *CSNK1e* in HLE and HLF cell lines (unpublished observation), implying that other genes modulated by HSF1 might eventually influence c-Myc activity in HCC cells.

Of note, we found that amplification of *c-Myc* and *HSF1* is a mutually exclusive event in human HCC specimens, suggesting that genetic alterations affecting one of the two genes are sufficient to activate the other one. In addition, the finding that half of the examined HCC (32 of 64, 50%) display amplification of either c-Myc or HSF1 implies that this genetic event is a very frequent alteration in human liver cancer. Of note, amplification (this study) and/or overexpression [15] of HSF1 (and c-Myc) occur especially in the most aggressive liver tumors, envisaging their crucial prognostic role in HCC.

The results from the present study might be clinically relevant. Indeed, on one hand our findings imply that *c-Myc*-overexpressing cells fully rely on HSF1 activity to exert their oncogenic potential. On the other hand, the present data strongly suggest that c-Myc cells might be specifically vulnerable to death induced by HSF1 inhibitors. Several of these drugs have been generated and exhibited a promising anti-neoplastic activity in preclinical models [33-35]. HSF1 inhibitors might be particularly important considering the biological aggressiveness of HCC with induction of c-Myc and HSF1, and the related unfavorable outcome of these patients. Thus, in the light of the difficulties to directly target c-Myc [11], the use of HSF1 inhibitors might represent a valid strategy for the treatment of HCC subset displaying activation of the c-Myc axis.

## MATERIALS AND METHODS

### Constructs and reagents

The constructs used in the experiments, including pT3EF1a-c-Myc, pT3-EF1α (empty vector), and pCMV/sleeping beauty transposase (SB), have been previously described [8]. The V5-tagged dominant negative form of human HSF1 (HSF1dn) [28] was cloned in a pT3-EF1α vector via the Gateway cloning strategy. Plasmids were purified using the Endotoxin-free Maxi Prep Kit (Sigma-Aldrich, St. Louis, MO, USA) before being injected into mice.

### Hydrodynamic injection, mouse monitoring

Hydrodynamic injection was performed as described previously [36]. In brief, 8μg pT3EF1a-c-Myc along with sleeping beauty transposase (SB) in a ratio of 25:1 were diluted in 2 ml saline (0.9% NaCl), then filtered through 0.22μm filter, and injected into the lateral tail vein of 6 to 8-week-old FVB/N mice in 5 to 7 seconds. To block the HSF1 signaling, high doses of HSF1dn (40μg) with low doses of pT3EF1a-c-Myc (8μg) were injected. Control mice were injected with pT3-EF1α (40μg). The care and use of mice for this study were carried out with the approval of the Institutional Animal Care and Use Committee (IACUC) of the University of California, San Francisco.

### Histology and immunohistochemistry

Liver lesions were fixed in 4% paraformaldehyde overnight at 4°C, embedded in paraffin, and evaluated by two board-certified pathologists (S.R. and F.D.) in accordance with the criteria by Frith et al [37]. For immunohistochemistry, antigen retrieval was performed in 10mM sodium citrate buffer (pH 6.0) by placement in a microwave on high for 10 min, followed by a 20 min cool down at room temperature. After a blocking step with 5% goat serum and Avidin-Biotin Blocking Kit (Vector Laboratories, Burlingame, CA), the slides were incubated with primary antibodies overnight at 4°C. Specifically, the anti-c-Myc (Santa Cruz Biotechnology Inc., Santa Cruz, CA, USA; # sc-40) and anti-HSF1 (Cell Signaling Technology Inc., Danvers, CA, USA; # 4356) primary antibodies were selected since they have been extensively validated by the manufacturers for immunohistochemistry. Slides were then subjected to 3% hydrogen peroxide for 10 min to quench endogenous peroxidase activity and subsequently the biotin conjugated secondary antibody was applied at a 1:500 dilution for 30 min at room temperature. The immunoreactivity was visualized with the Vectastain Elite ABC kit (Vector Laboratories), using Vector NovaRED™ (Vector Laboratories) as the chromogen. Slides were counterstained with Mayer’s hematoxylin.

### Western blot analysis

HCC cell line extracts and mouse livers tissues were homogenized in lysis buffer [30 mM Tris (pH 7.5), 150 mM NaCl, 1% NP-40, 0.5% Na deoxycholate, 0.1% SDS, 10% glycerol and 2mM EDTA] containing the Complete Protease Inhibitor Cocktail (ThermoFisher Scientific, Waltham, MA, USA). Protein concentrations were determined with the Bio-Rad Protein Assay Kit (Bio-Rad, Hercules, CA, USA) using bovine serum albumin as standard. For Western blot analysis, aliquots of 40 μg were denatured by boiling in Tris-Glycine SDS Sample Buffer (Bio-Rad), separated by SDS-PAGE, and transferred onto nitrocellulose membranes (Bio-Rad) by electroblotting. Membranes were blocked in Pierce Protein-free Tween 20 Blocking Buffer (ThermoFisher Scientific) for 1 h and probed with the following specific antibodies: anti-HSF1 (# 4356), anti-SCD1 (# 2438), anti-ACAC (# 3662), anti-DDK (# 14793), anti-HKII (# 2867), anti-LDHA/C (Cell Signaling Technology Inc.; # 3558), anti-ODC (# sc-390366), anti-NRF1 (Santa Cruz Biotechnology; # sc-365651), anti-c-Myc (# sc-40), and anti-FASN (Bethyl Laboratories Inc., Montgomery, TX, USA; # A301-324A). Anti-β-Actin (# A5441; Sigma-Aldrich) was used as loading control. Each primary antibody was followed by incubation with horseradish peroxidase-secondary antibody (Jackson ImmunoResearch Laboratories Inc., West Grove, PA, USA) diluted 1:5000 for 30 min and proteins were revealed with the Super Signal West Femto (Pierce Chemical Co., New York, NY, USA).

### *In vitro* studies

The human HLE, HLF, and MHCC97-L HCC cell lines, after validation (Genetica DNA Laboratories, Burlington, NC, USA), were used in this study. Cells were grown in a 5% CO_2_ atmosphere, at 37°C, in RPMI Medium supplemented with 10% fetal bovine serum (FBS; Gibco, Grand Island, NY, USA) and penicillin/streptomycin (Gibco). For knockdown studies, HLE and HLF cells were transfected with 50nM siRNA targeting human *HSF1* (ID # L-012109-02-0005; GE, Dharmacon, Lafayette, CO, USA) or *c-Myc* (ID # S9129; Life Technologies, Grand Island, NY) in the Lipofectamine RNAiMax Transfection Reagent (Life Technologies). The HCC3-4 and HCC4-4 mouse HCC cell lines, isolated from c-Myc mouse liver tumors [29], were kindly provided by Dr. Dean W. Felsher from Stanford University (Stanford, CA, USA) and were confirmed to express elevated levels of c-Myc (not shown). The mouse cell lines were transfected with 50nM siRNA targeting mouse *HSF1* (ID # 15499; GE, Dharmacon) or human *c-Myc* gene. A scramble small interfering RNA (siRNA; ID # 4390846; Life Technologies) was used as negative control. Transient transfection experiments with DDK-tagged *HSF1* (ID # RC200314) or untagged *c-Myc* (ID # SC112715) cDNA in pCMV6-Entry and pCMV6-XL5 plasmid (OriGene Technologies, Rockville, MD, USA), respectively, were conducted in the MHCC97-L HCC cell line using the Lipofectamine 2000 Reagent (Life Technologies) following the manufacturer’s protocol. The HSF1inhibitor, KRIBB11 (Sigma-Aldrich; final concentration 20 μM) was administered to HLE, HLF, HCC3-4, and HCC4-4 cell lines for 48 h after 24h serum deprivation. RNA was extracted 48 hours after siRNA silencing, cDNA transfection, and KRIBB11 treatment. Cell proliferation and apoptosis were determined in human and mouse HCC cell lines at 24- and 48-hour timepoints using the BrdU Cell Proliferation Assay Kit (Cell Signaling Technology Inc.) and the Cell Death Detection Elisa Plus Kit (Roche Molecular Biochemicals, Indianapolis, IN, USA), respectively, following the manufacturers’ instructions. All experiments were repeated at least three times in triplicate.

### Quantitative reverse transcription real-time polymerase chain reaction (qRT-PCR)

Validated Gene Expression Assays for human *HSF1* (ID # Hs00232134_m1), *c-Myc* (ID # Hs00153408_m1), *ODC* (ID # Hs00159739_m1), *LDHA* (ID # Hs01378790_g1), and *β-Actin* (ID # 4333762T) genes as well as for mouse *HSF1* (ID # Mm01201402_m1) and *β-Actin* (ID # Mm00607939_s1) genes were purchased from Applied Biosystems (Foster City, CA, USA). PCR reactions were performed with 100 ng of cDNA of the collected samples or cell lines, using an ABI Prism 7000 Sequence Detection System with TaqMan Universal PCR Master Mix (Applied Biosystems). Cycling conditions were: denaturation at 95°C for 10 min, 40 cycles at 95°C for 15 s, and then extension at 60°C for 1 min. Quantitative values were calculated by using the PE Biosystems Analysis software and expressed as N target (NT). NT = 2^-ΔCt^, wherein ΔCt value of each sample was calculated by subtracting the average Ct value of the target gene from the average Ct value of the *β- Actin* gene.

### Assessment of HSF1 activity, fatty acid synthesis, total cholesterol and triglyceride content, and lactate dehydrogenase activity

HSF1 activity in mouse frozen liver tissues was determined using the HSF1 ELISA kit (Enzo Life Sciences, Farmingdale, NY), following the manufacturers’ protocol. Fatty acid synthesis was measured by incorporation of [U-^14^C] acetate into lipids as described [38]. Briefly, lipids were Folch extracted and counted for ^14^C. Total cholesterol and triglyceride levels in mouse liver specimens were assessed using the Cholesterol Quantitation Kit and the Triglyceride Quantification Kit (BioVision Inc., Mountain View, CA, USA), respectively, following the manufacturer’s recommendation. Lactate dehydrogenase (LDH) activity was assessed using the LDH activity assay (BioVision Inc.) according to the manufacturer’s instructions.

### Human tissue samples

A collection of sixty-four frozen and corresponding formalin-fixed paraffin-embedded HCC specimens from previous investigations [12, 15] were used. Tumors were divided in HCC with shorter survival/poorer outcome (HCCP; *n* = 32) and longer survival/better outcome (HCCB; *n* = 32) survival, characterized by < 3 and > 3 years’ survival following partial liver resection, respectively. HCC specimens were collected at the Medical Universities of Greifswald (Greifswald, Germany) and Sassari (Sassari, Italy). Institutional Review Board approval was obtained at the local Ethical Committee of the Medical Universities of Greifswald and Sassari. Informed consent was obtained from all individuals.

### Copy number variation (CNV) assay

DNA from 64 formalin-fixed paraffin-embedded tissues was extracted using the Qiagen Tissue kit (Qiagen, Valencia, CA, USA). DNA CNV levels were quantified using the real-time fluorescence detection method. The protocol was optimized for relative quantification of DNA copy number in the human genome and the reaction was performed on Rotor-Gene 6000 (Qiagen) according to the Type-it CNV Probe PCR + qCKit handbook. For the gene of interest (GOI), we used the provided Type-it CNV Reference Probe Assay by duplex PCR for reliable ΔΔCT-based quantification of the CNV. The comparative or ΔΔCT method of relative quantification relies on direct comparison of CT values of the target sample and a calibrator or control (reference) sample. Reference assay was labeled with MAX dye, which was detected on the HEX/VIC channel. For the *HSF1* gene, the TaqMan Copy Number Assay (ID # Hs03673241_cn; ThermoFisher Scientific) was used, and probe was labeled with FAM dye, which was detected on the FAM/GREEN channel by duplex PCR. The validation of the internal reference and HSF1 assay was performed using the standard curve method of absolute quantitation with normalization to the internal reference for copy number. Copy number data analysis was performed by Rotor-Gene Q Series Software (Qiagen). Reaction setup for duplex PCR was performed using DNA 20 ng of template DNA. Amplification of HSF1 was defined as a HSF1/Reference ratio of 1.5 or higher.

### Fluorescence *in situ* hybridization

Fluorescence in situ hybridization (FISH) analysis was performed by using bacterial artificial chromosome (BAC) probes, covering the c-Myc (RP11-440N18) locus at the 8q24.21 genomic region and a reference locus (CEP7) at the 7q11.23 chromosome as control, onto 4-micron sections of formalin-fixed paraffin-embedded tissue from each tumor (*n* = 64). The BAC clones were chosen according to their genomic location as defined in the UCSC genome browser (http://genome.ucsc.edu) and were obtained from ThermoFisher Scientific. The BAC DNA was isolated according to the manufacturer’s instructions, labeled with different fluorochromes by nick translation, denatured, and hybridized to pretreated slides. Slides were then incubated, washed, and mounted with DAPI in an antifade solution. Two hundred interphase nuclei were counted from each tumor by using an Olympus BX61 microscope (Olympus Life Science, Waltham, MA, USA), equipped with an Imaging QICAM controlled by Genikon software (Nikon, Melville, NY, USA). Amplification of c-Myc was defined as a Myc/CEP7 ratio of 1.5 or higher.

### Statistical analysis

GraphPad Prism version 6.0 (GraphPad Software Inc., La Jolla, CA, USA) was used to evaluate statistical significance by Tukey-Kramer, Student’s *t* and Mann-Whitney tests and linear regression analyses. Values of *P* < 0.05 were considered significant. Data are expressed as mean ± standard deviation for each group. Two-tailed unpaired *t* test was used to compare the differences between two groups.

## SUPPLEMENTARY MATERIALS FIGURES



## Abbreviations

ACAC: acetyl-CoA carboxylase
CE: cholesterol
CNV: copy number variation
FASN: fatty acid synthase
FISH: fluorescence in situ hybridization
HCC: hepatocellular carcinoma
HCCB: hepatocellular carcinoma with better prognosis/longer survival
HCCP: hepatocellular carcinoma with poorer prognosis/shorter survival
HKII: hexokinase II
HSF1: heat shock transcription factor 1
LDHA/C: lactate dehydrogenase A/C
mTOR: mammalian target of rapamycin
mTORC: mTOR complex
NRF1: nuclear respiratory factor 1
ODC: ornithine decarboxylase
SCD1: stearoyl-CoA desaturase 1
siRNA: small interfering RNA
TFAM: transcription factor A, mitochondrial
TG: triglycerides
