# Supplementary material for: Deregulated c-Myc requires a functional HSF1 for experimental and human hepatocarcinogenesis
Source: Oncotarget. 2017 Oct 3;8(53):90638–50. doi: 10.18632/oncotarget.21469 (PMC5710874; doi:10.18632/oncotarget.21469)
Supplement: Supplementary file 1 [file oncotarget-08-90638-s001.pdf]

Deregulated c-Myc requires a functional HSF1 for experimental and human hepatocarcinogenesis

SUPPLEMENTARY MATERIALS

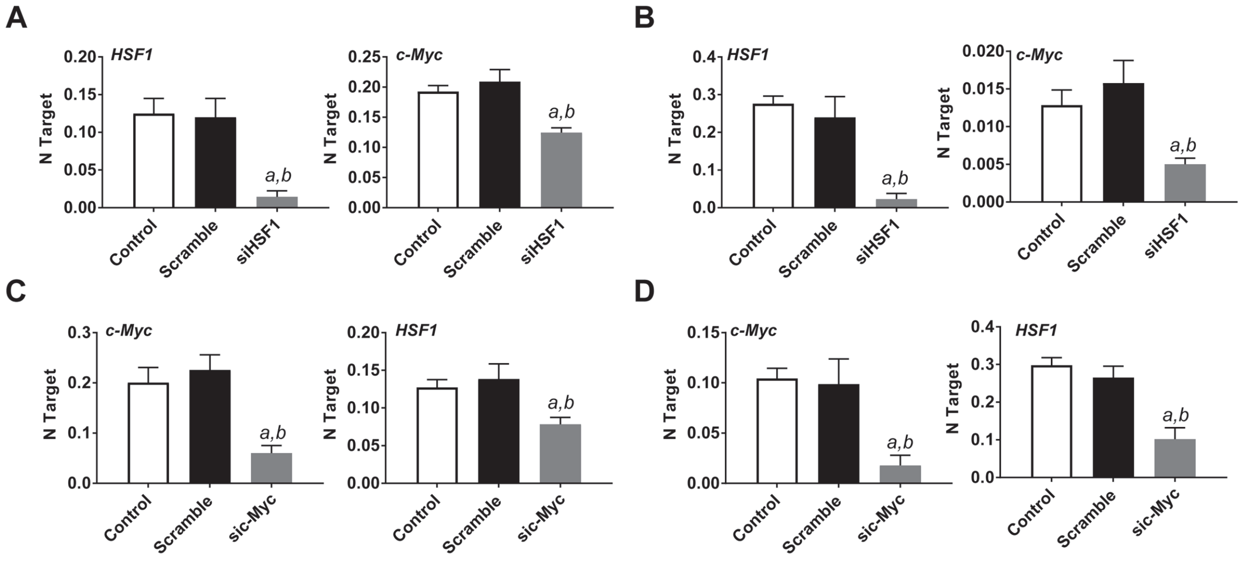

Supplementary Figure 1: Reciprocal regulation of HSF1 and c-Myc in human HLE and HLF HCC cell lines as detected by reverse transcription real-time PCR. (A) Suppression of *HSF1* expression by specific siRNA for 48h induces downregulation of *c-Myc* in HLE cells. (B) Equivalent results were obtained in HLF cells. (C) Knockdown of *c-Myc* by siRNA triggers downregulation of *HSF1* in HLE cells. (D) Equivalent results were obtained in HLF cells. Number target (NT) = $2^{-\Delta Ct}$, wherein ΔCt value of each sample was calculated by subtracting the average Ct value of the *HSF1* or *c-Myc* gene from the average Ct value of the *β-actin* gene. Tukey–Kramer test: P <0.0001 *a*, vs control (untreated cells); *b*, vs scramble siRNA.

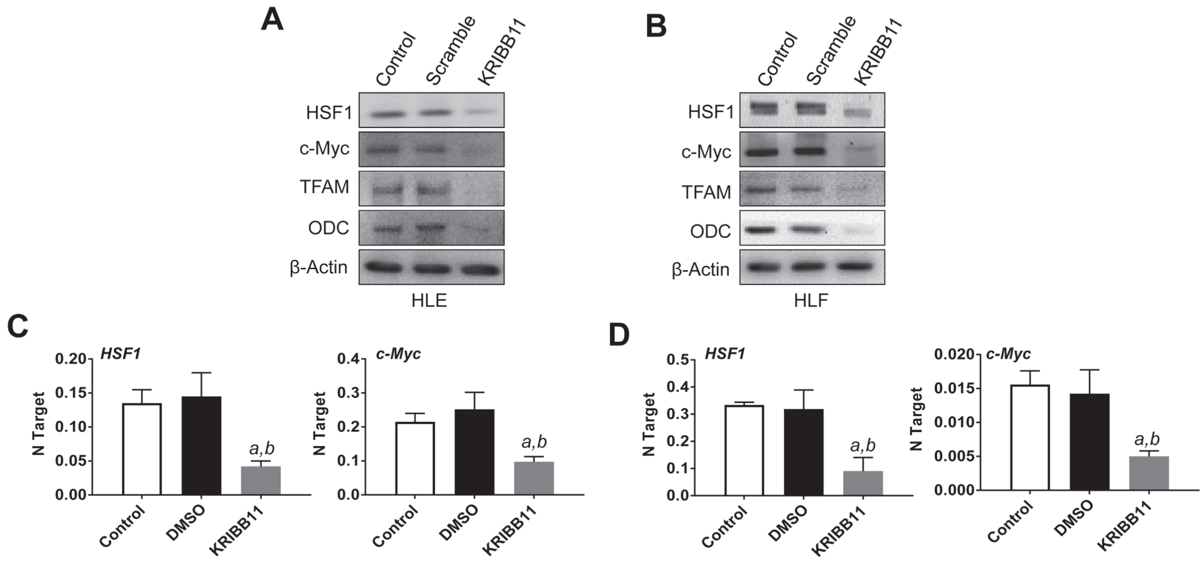

Supplementary Figure 2: The HSF1 inhibitor KRIBB11 negatively regulates c-Myc in human HLE and HLF HCC cell lines. Treatment with KRIBB11 (20µM) for 48h induces downregulation of HSF1, c-Myc, TFAM, and ODC proteins in HLE (A) and HLF (B) cells, as detected by Western blotting. Equivalent results for *HSF1* and *c-Myc* were obtained at mRNA level by reverse transcription real-time PCR in HLE (C) and HLF (D) cells. Number target (NT) = $2^{-\Delta Ct}$, wherein ΔCt value of each sample was calculated by subtracting the average Ct value of the *HSF1* or *c-Myc* gene from the average Ct value of the *β-actin* gene. Tukey–Kramer test: *P* <0.0001 *a*, vs control (untreated cells); *b*, vs DMSO (solvent).

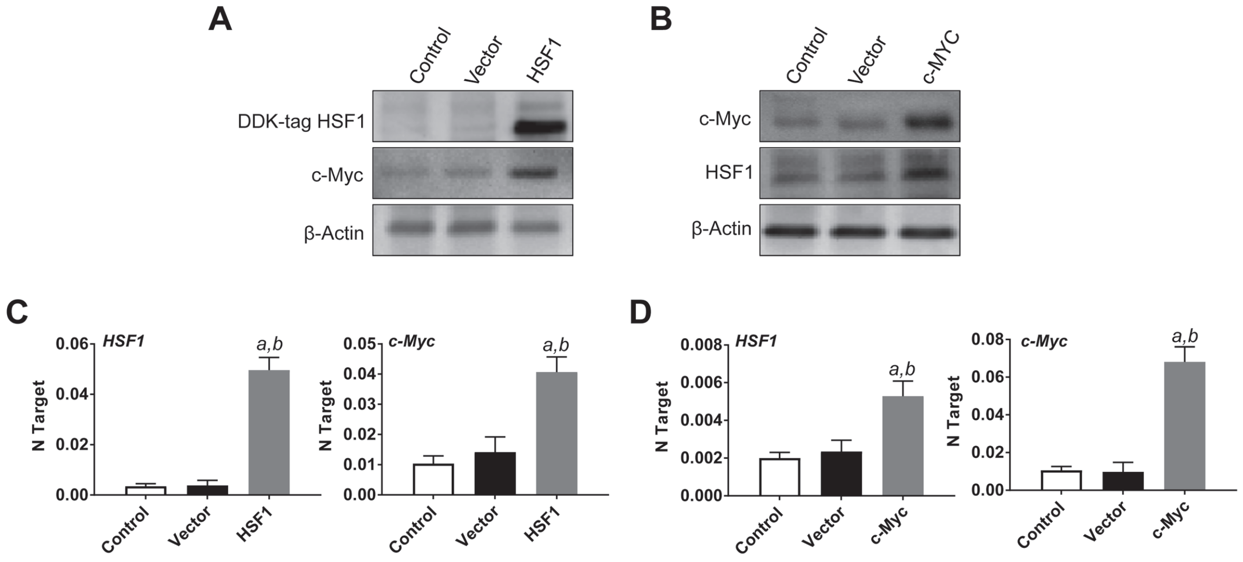

Supplementary Figure 3: Reciprocal regulation of HSF1 and c-Myc following HSF1 and c-Myc in the human MHCC97-L HCC cell line. Transient transfection for 48h with plasmids encoding *HSF1* (A,C) and *c-Myc* (B,D) leads to upregulation of HSF1 and c-Myc, as detected by Western blotting (A,B) and reverse transcription real-time PCR (C,D). Number target (NT) = $2^{-\Delta Ct}$, wherein ΔCt value of each sample was calculated by subtracting the average Ct value of the *HSF1* or *c-Myc* gene from the average Ct value of the *β-actin* gene. Tukey–Kramer test: *P* <0.0001 *a*, vs control (untreated cells); *b*, vs DMSO (solvent).

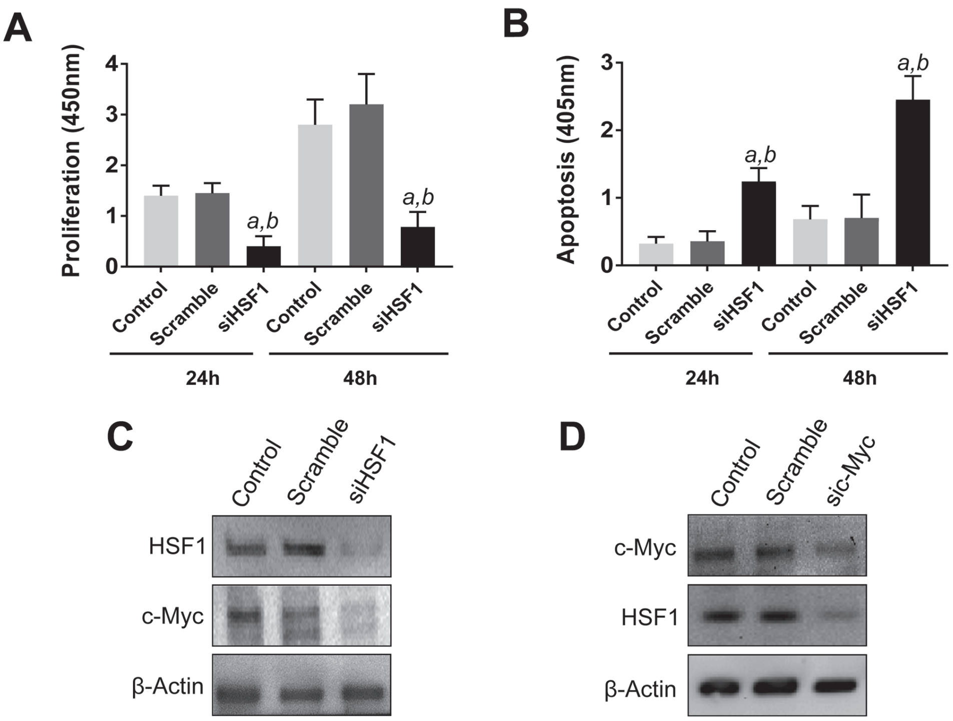

Supplementary Figure 4: Reciprocal regulation of HSF1 and c-Myc and growth restraint following HSF1 silencing in the mouse HCC4-4 c-Myc cell line. (A,B) Suppression of *HSF1* expression by specific siRNA for 24 and 48h results in decreased proliferation and augmented apoptosis. Tukey–Kramer test: P <0.0001 *a*, vs control (untreated cells); *b*, vs scramble siRNA. (C) At the molecular level, HSF1 silencing leads to c-Myc downregulation. Effect at 48h after siRNA is shown. (D) Conversely, knockdown of c-Myc by siRNA triggers downregulation of HSF1. β-Actin was used as a loading control.

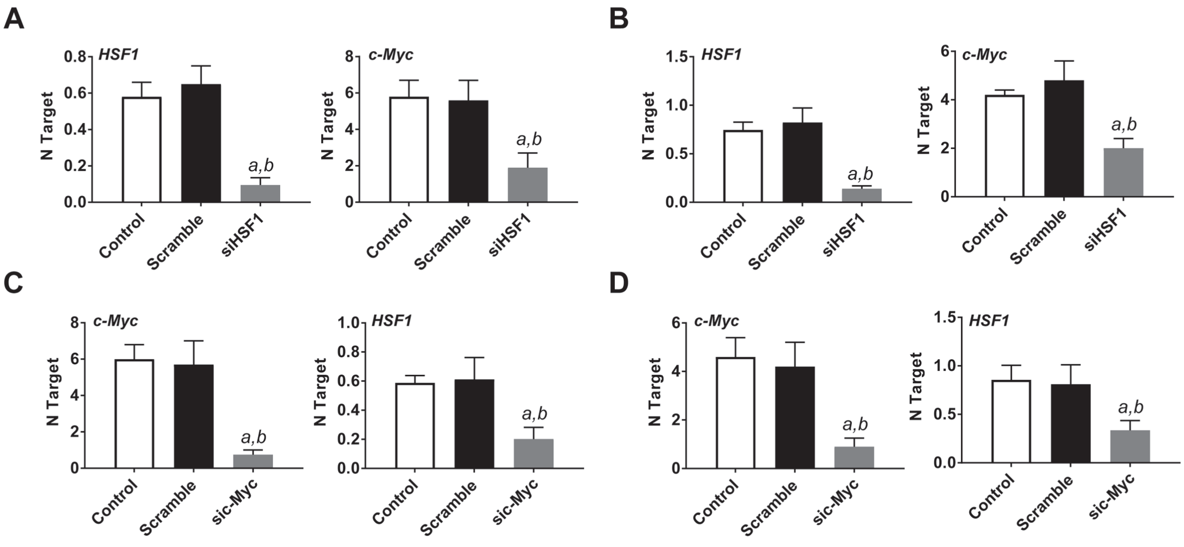

Supplementary Figure 5: Reciprocal regulation of HSF1 and c-Myc in mouse HCC3-4 and HCC4-4 cell lines as detected by reverse transcription real-time PCR. (A) Suppression of *HSF1* expression by specific siRNA for 48h induces downregulation of *c-Myc* in HCC3-4 cells. (B) Equivalent results were obtained in HCC4-4 cells. (C) Knockdown of *c-Myc* by siRNA triggers downregulation of HSF1 in HCC3-4 cells. (D) Equivalent results were obtained in HCC4-4 cells. Number target (NT) = $2^{-\Delta Ct}$, wherein ΔCt value of each sample was calculated by subtracting the average Ct value of the *HSF1* or *c-Myc* gene from the average Ct value of the *β-actin* gene. Tukey–Kramer test: P <0.0001 *a*, vs control (untreated cells); *b*, vs scramble siRNA.

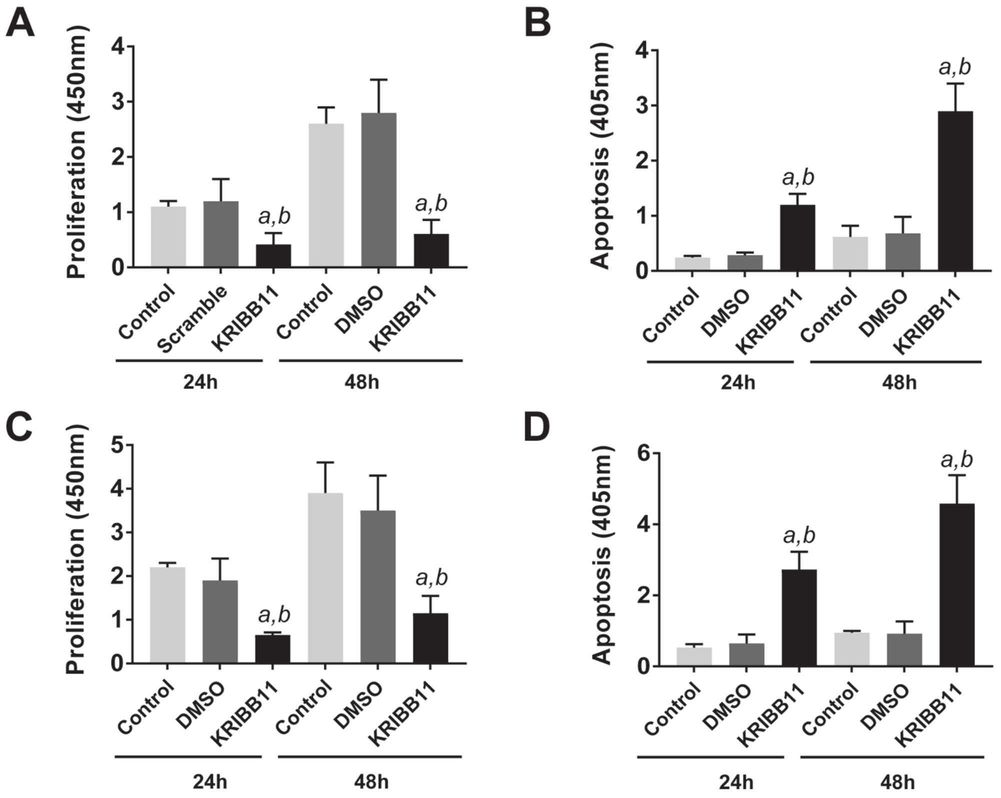

Supplementary Figure 6: The HSF1 inhibitor KRIBB11 induces growth restraint of mouse HCC3-4 and HCC4-4 c-Myc cell lines. Treatment with KRIBB11 (20μM) for 24h and 48h results in decreased proliferation (A) and augmented apoptosis (B) in HCC3-4 cells. Equivalent results for proliferation (C) and apoptosis (D) were obtained in HCC4-4 cells. Tukey–Kramer test: P <0.0001 *a*, vs control (untreated cells); *b*, vs DMSO (solvent).
